# Database search of spontaneous reports and pharmacological investigations on the sulfonylureas and glinides-induced atrophy in skeletal muscle

**DOI:** 10.1002/prp2.28

**Published:** 2014-03-03

**Authors:** Antonietta Mele, Sara Calzolaro, Gianluigi Cannone, Michela Cetrone, Diana Conte, Domenico Tricarico

**Affiliations:** 1 Departments of Pharmacy-Drug Science, University of BariBari, Italy; 2 Departments of Pharmacovigilance, University-Hospital Policlinico, Ministry of HealthBari, Italy

**Keywords:** Atrophy, oral antidiabetic drugs, pharmacovigilance, skeletal muscle

## Abstract

The ATP-sensitive K^+^ (KATP) channel is an emerging pathway in the skeletal muscle atrophy which is a comorbidity condition in diabetes. The “in vitro” effects of the sulfonylureas and glinides were evaluated on the protein content/muscle weight, fibers viability, mitochondrial succinic dehydrogenases (SDH) activity, and channel currents in oxidative soleus (SOL), glycolitic/oxidative flexor digitorum brevis (FDB), and glycolitic extensor digitorum longus (EDL) muscle fibers of mice using biochemical and cell-counting Kit-8 assay, image analysis, and patch-clamp techniques. The sulfonylureas were: tolbutamide, glibenclamide, and glimepiride; the glinides were: repaglinide and nateglinide. Food and Drug Administration-Adverse Effects Reporting System (FDA-AERS) database searching of atrophy-related signals associated with the use of these drugs in humans has been performed. The drugs after 24 h of incubation time reduced the protein content/muscle weight and fibers viability more effectively in FDB and SOL than in the EDL. The order of efficacy of the drugs in reducing the protein content in FDB was: repaglinide (EC50 = 5.21 × 10^−6^) ≥ glibenclamide(EC50 = 8.84 × 10^−6^) > glimepiride(EC50 = 2.93 × 10^−5^) > tolbutamide(EC50 = 1.07 × 10^−4^) > nateglinide(EC50 = 1.61 × 10^−4^) and it was: repaglinide(7.15 × 10^−5^) ≥ glibenclamide(EC50 = 9.10 × 10^−5^) > nateglinide(EC50 = 1.80 × 10^−4^) ≥ tolbutamide(EC50 = 2.19 × 10^−4^) > glimepiride(EC50=–) in SOL. The drug-induced atrophy can be explained by the KATP channel block and by the enhancement of the mitochondrial SDH activity. In an 8-month period, muscle atrophy was found in 0.27% of the glibenclamide reports in humans and in 0.022% of the other not sulfonylureas and glinides drugs. No reports of atrophy were found for the other sulfonylureas and glinides in the FDA-AERS. Glibenclamide induces atrophy in animal experiments and in human patients. Glimepiride shows less potential for inducing atrophy.

## Introduction

Muscle atrophy, or muscle wasting, results from loss of muscle protein. Sedentary lifestyle is a cause of muscle atrophy (Fanzani et al. 2012). Common causes include neuromuscular diseases, such as spinal cord atrophy, multiple sclerosis, amyotrophic lateral sclerosis, or Guillain–Barre syndrome. Diabetic neuropathy and nerve damage associated with diabetes may also lead to atrophy of the muscles (Bonaldo and Sandri 2013). Hyperglycemia or diabetes are often comorbidity conditions in patients affected by neuromuscular disorders; hyperglycemia, per se, may cause muscle atrophy. Muscle atrophy is a rare adverse reaction associated with a drug treatment. Confounding factors for identifying this drug-signal are comorbidity conditions associated with atrophy or the polytherapy. Some drugs such as the antivirals and glucocorticoids are known to cause muscle atrophy in animal and human experiments (Valiyil and Christopher-Stine 2010). Glucocorticoid myopathy is often characterized by muscular atrophy, which is believed to be due to suppressed protein synthesis and growth, enhanced proteolysis, and apoptosis induction (Dirks-Naylor and Griffiths 2009).

ATP-sensitive K^+^ (KATP) channel blockers are prescribed to individuals who are in the diabetes-aged population and who display impaired glucose-induced insulin release (Gribble and Reimann 2003; Arnoux et al. 2010; Ashcroft 2010). This channel is a complex that is composed of the sulfonylureas receptor type-1 or type 2A/B (SUR1, SUR2A, SUR2B) and the inwardly rectifying K^+^ channel (Kir6.2) subunits. The SUR subunits carry the binding sites for the KATP channel openers and blockers (Babenko et al. 2000).

The sulfonylureas and glinides are being investigated for use in the treatment of hypotension that results from septic shock, ischemic trauma, and neonatal diabetes (Jahangir and Terzic 2005; Koster et al. 2005; Flechtner et al. 2006; Pearson et al. 2006; Flanagan et al. 2007; Mlynarski et al. 2007; Simard et al. 2008; Ashcroft 2010).

These drugs belong to the following distinct classes: the first-generation sulfonylureas (chlorpropamide, tolbutamide, and tolazamide), which are low-affinity ligands of the SUR1 subunit that cause the release of insulin at micromolar concentrations; the second- (glipizide and glyburide) and third-generation sulfonylureas (glimiperide and acetohexamide) which are high-affinity and slowly reversible ligands of the SUR1 subunit that act at nanomolar concentrations and the glinides, which lack the sulfonylurea moiety and include repaglinide, nateglinide, and meglitinide, which are high-affinity SUR1 ligands. These drugs exhibit a rapid onset/offset action compared with that of the sulfonylureas (Gribble and Reimann 2003).

However, severe hypoglycemia, weight gain, and cardiovascular side effects limit their use in special populations (Zünkler 2006). In addition, therapeutic concentrations of sulfonylureas and high doses of glinides induce the apoptosis of beta cells, beta cell lines, or cell lines that express the recombinant KATP channel subunits, and these effects are mediated by SUR1 (Maedler et al. 2005; Hambrock et al. 2006). Long-term exposure of beta cells to sulfonylureas induces a reduction in the insulin content and the number of KATP channels (Takahashi et al. 2007). It has been proposed that sulfonylurea-induced atrophy contributes to the loss of beta cell mass that characterizes the progression of diabetes (Takahashi et al. 2007).

Skeletal muscle side effects were reported for glibenclamide and glipizide that show arthralgia, myalgia, and leg cramp, however, in less than 3% of the patients, while no muscular effects were reported for chlorpropamide, tolbutamide and glimepiride. A higher incidence of musculoskeletal effects was observed with repaglinide and nateglinide who show arthralgia and back pain in 4–6% of the patients as reported in Micromedex 2.0 2012. In animal experiments, downregulation of the Kir6.2 and SUR1 subunits composing the KATP channels is associated with muscle atrophy of the slow-twitch soleus (SOL) muscle in 14-day-hindlimb-unloaded rats, an animal model of muscle disuse. The long-term incubation of the control muscles in vitro with the KATP channel blocker glibenclamide (10^−6^ mol/L) reduced the KATP currents with atrophy and these effects were prevented by the KATP channel opener diazoxide (10^−4^ mol/L) (Tricarico et al. 2010). More recently, we have shown that the long-term incubation of the fibers with an antibody targeting the pyruvate kinase enzyme, which is functionally coupled to the Kir6.2 subunit, reduced the KATP current reducing the diameter with atrophy. Therefore, it seems that the “in vivo” downregulation of the KATP channel subunits or their “in vitro” pharmacological blockade activates atrophic signaling in skeletal muscle (Mele et al. 2012). As opposite, the activation of the KATP channels may lead to cytoprotection (Teshima et al. 2003; Jahangir and Terzic 2005).

In the present work, the “in vitro” effects of the sulfonylureas and glinides were evaluated on the protein content/muscle weight ratio and fibers viability used as indexes of atrophy, mitochondrial succinic dehydrogenases (SDH) activity and channel currents in slow-twitch oxidative SOL, fast-twitch glycolitic/oxidative flexor digitorum brevis (FDB) and fast-twitch glycolitic extensor digitorum longus (EDL) muscle fibers of mice. The sulfonylureas were: tolbutamide, glibenclamide, and glimepiride; the glinides were: repaglinide and nateglinide. Experiments were performed using biochemical and cell-counting Kit-8 assay, imagine analysis, and patch-clamp techniques. The role of the KATP channel subunits in the cytotoxicity induced by the drugs was investigated in HEK293 cell line expressing the Kir6.2 subunit or the combination of Kir6.2+SUR1 and Kir6.2+SUR2A subunits and in not transfected cells.

The Food and Drug Administration-Adverse Effects Reporting System (FDA-AERS) database searching of atrophy-related signals associated with the use of the sulfonylureas and glinides in humans has been performed. This database is a passive surveillance system that accepts spontaneous reports of adverse events following any US licensed drugs from providers, health care workers, and the public. Although AERS cannot usually prove causal associations between drugs and adverse events and do not give the incidence, it can detect signals to be tested with more rigorous methods including an experimental one.

## Materials and Methods

### Drugs and solutions

The drugs under investigation were: tolbutamide (tolb.), glibenclamide/glyburide (glib.), glimepiride (glimep.), repaglinide (repa.), and nateglinide (nate.) (10^−7^ to 500 × 10^−6^ mol/L), 5-hydroxydecanoate (5-HD) (10^−7^ to 500 × 10^−6^ mol/L), diazoxide (diazo.) (250 × 10^−6^ mol/L), and staurosporine (2 × 10^−6^ mol/L) as apoptotic agent.

IUPAC Name: (5-chloro-N-[2-[4-(cyclohexylcarbamoylsulfamoyl)phenyl]ethyl]-2- methoxybenzamide (glibenclamide/glyburide); 4-ethyl-3-methyl-N-[2-[4-[(4-methylcyclohexyl)carbamoylsulfamoyl]phenyl]ethyl]-5-oxo-2H-pyrrole-1-carboxamide (glimepiride); 1-butyl-3-(4-methylphenyl)sulfonylurea (tolbutamide); 5-hydroxydecanoate (5-HD); 8,12-Epoxy-1H,8H-2,7b,12a-triazadibenzo(a,g)cyclonona(cde)trinden-1-one,2,3,9,10,11,12-hexahydro-9-methoxy-8-methyl-10-(methylamino)-,(8alpha,9beta,10beta,12alpha)-(+)- (staurosporine); 2-ethoxy-4-[2-[[(1S)-3-methyl-1-(2-piperidin-1-ylphenyl)butyl]amino]-2-oxoethyl]benzoic acid (repaglinide); (2R)-3-phenyl-2-[(4-propan-2-ylcyclohexanecarbonyl)amino]propanoic acid (nateglinide); 7-chloro-3-methyl-4H-1*λ*^6^,2,4-benzothiadiazine 1,1-dioxide (diazoxide).

The drug concentrations tested (10^−7^ to 500 × 10^−6^ mol/L) in our experiments were selected on the basis on their potency as KATP channel blockers in native skeletal muscle and on the plasma glibenclamide concentrations (Groop et al. 1991; Tricarico et al. 2006). The plasma drug concentration of glibenclamide in human healthy volunteers is between 100–200 × 10^−9^ mol/L corresponding to an oral dose ≤10 mg (Groop et al. 1991). From our calculation, the corresponding mass dose in mice is 1 mg/kg (0.03 mg/mouse of 30 g body weight) based on the body surface area (BSA) conversion (Reagan-Shaw et al. 2008). Taking into account a circulating blood volume of about 2.6 mL per mouse (Diehl et al. 2001), the calculated circulating concentration of glibenclamide is about 23 × 10^−6^ mol/L in this animal species which is in the range of the concentrations tested in our experiments (0.1 × 10^−6^ to 100 × 10^−6^ mol/L) on muscle fibers. The muscles and the isolated fibers were incubated with the Dulbecco's modified Eagle's medium (DMEM+) solution composed by 1× antibiotics (1%), l-glutamine (1%), FBS (10%) in the presence or absence of aliquots of the stock solution of the drugs to final concentrations ranging between 0.00005 and 0.25 mg/mL. Stock solutions (1 mol/L) of these drugs were prepared in dimethyl sulfoxide (DMSO). The final DMSO concentration in the DMEM+ medium did not exceed 0.05%. The normal Ringer solution used during muscle biopsy and for preparation of isolated fibers contained: 145 × 10^−3^ mol/L NaCl, 5 × 10^−3^ mol/L KCl, 1 × 10^−3^ mol/L MgCl_2_, 0.5 × 10^−3^ mol/L CaCl_2_, 5 × 10^−3^ mol/L glucose, and 10 × 10^−3^ mol/L 3-(*N-*morpholino) propanesulfonate (Mops) sodium salt and was adjusted to pH 7.2 with Mops acid.

For fiber viability experiments, the isolated fibers were superfused with a bath solution that contained: 143 × 10^−3^ mol/L NaCl, 5.4 × 10^−3^ mol/L KCl, 1.8 × 10^−3^ mol/L CaCl_2_, 0.33 × 10^−3^ mol/L NaH_2_PO_4_, 0.5 × 10^−3^ mol/L MgCl_2_, and 5 × 10^−3^ mol/L HEPES, adjusted to pH 7.4 with NaOH.

For patch-clamp experiments, the pipette solutions contained: 150 × 10^−3^ mol/L KCl, 2 × 10^−3^ mol/L CaCl_2_, and 1 × 10^−2^ mol/L Mops (pH 7.2). The bath solution contained: 150 × 10^−3^ mol/L KCl, 5 × 10^−3^ mol/L EGTA, and 1 × 10^−2^ mol/L Mops (pH 7.2). K_2_ATP (100 × 10^−6^ mol/L to 5 × 10^−3^ mol/L) was added to the bath solution as requested. Stock solutions of diazo (5 × 10^−2^ mol/L), sulfonylureas, and glinides (6 × 10^−3^ mol/L) were prepared by dissolving the drugs in DMSO. Microliter amounts of the drug stock solution were then added to the bath solution. DMSO applied at the maximal concentration tested, which was 0.05%, did not affect the channel currents in the absence or in the presence of ATP (solvent control). All chemicals including drugs were purchased from Sigma-Aldrich (St. Louis, MO).

### Drug-dependent atrophy in different skeletal muscle phenotypes of mice

Experiments were approved by the Italian Health Department (Art. 9 del Decreto Legislativo 116/92: Decreto no. 33/2000-B del Dipartimento degli Alimenti e Nutrizione e della Sanita' Pubblica) and performed under the supervision of a local veterinary official. Slow-twitch SOL, fast-twitch EDL and the fast-twitch FDB muscles were removed from male mice (body weight = 20–26 g) sacrificed by cervical dislocation.

Intact muscles used for visual and stereo microscope inspection were carefully pinned on Petri disks. Muscles damaged during dissection or showing contraction during the incubation period were discarded. All experiments were performed under 5% CO_2_–95% O_2_ atmosphere for the maintenance of aerobic conditions, at 37°C, and the muscles were incubated for 1, 6, 24, and 48 h with the drug solution under investigation. At the end of the incubation period, all muscle samples were blotted on absorbent paper, carefully weighed, and rapidly frozen in liquid nitrogen. Muscles were incubated with DMEM+ solution and enriched with the sulfonylureas and glinides (10^−7^ mol/L to 5 × 10^−4^ mol/L) or with sulfonylureas and glinides + diazoxide (250 × 10^−6^ mol/L). The observed values of total proteins and wet weight of the muscles were compared with those of the corresponding contralateral muscles from the same animal incubated with DMEM+ solution alone. Samples from muscle homogenates were used for total protein content quantification using Bio-Rad protein assay (Bio-Rad Labs GmbH, München, Germany). Some muscles were used for fiber viability investigation. Single fibers were obtained by enzymatic dissociation as previously described (Tricarico et al. 2006).

### Fiber morphology measurements and viability

Blind evaluation of the morphological parameters of the isolated fibers was performed in the control condition (DMEM^+^ solution) and in the presence of different drugs (DMEM^+^ + drugs) under investigation. The fibers morphology and vitality were evaluated by a direct visual inspection and cell counting using QuantiCell 900 integrated imaging system (VisiTech International Ltd, Sunderland, UK). Briefly, isolated fibers, before analysis, were equilibrated in DMEM (300 mOsmol/L) for 15 min at 25°C. Cell's death was defined as cells showing marked changes of >40% in morphology such as length and diameter; and failing contractility response following ATP (1 × 10^−3^ mol/L) or KCl (15 × 10^−3^ mol/L) addition to the bath solution. The isolated cells superfused with a bath solution (143 × 10^−3^ mol/L NaCl, 5.4 × 10^−3^ mol/L KCl, 1.8 × 10^−3^ mol/L CaCl_2_, 0.33 × 10^−3^ mol/L NaH_2_PO_4_, 0.5 × 10^−3^ mol/L MgCl_2_, and 5 × 10^−3^ mol/L HEPES, adjusted to pH 7.4 with NaOH) were placed in an experimental chamber (400 *μ*L volume) placed on the stage of an inverted Eclipse TE300 microscope with an 40× Plan-Fluor objective (Nikon, Firenze, Italy) and linked at the center pore to an electrovalves controlled perfusion system assuring a constant gravity-driven flow rate of 4 mL/min (Warner Instr., Hamden, CT). A digital image for each fiber or plate was stored for further evaluation. For each fiber, three individual measurements were performed at three different points. The appearance of multiple sarcolemma blebs often preceded cellular death.

### Patch-clamp experiments

Experiments were performed in inside out configurations by using the standard patch-clamp technique. Channel currents were recorded during voltage steps going from 0 mV of holding potential to −60 mV voltage membrane (Vm) immediately after excision, at 20–22°C, in the presence of 150 × 10^−3^ mol/L KCl on both sides of membrane patches in the absence (control) or presence of ATP (0.1–5 × 10^−3^ mol/L) in the bath. The currents were recorded at a 1-kHz sampling rate (filter = 0.2 kHz) by using an Axopatch-1D amplifier equipped with a CV-4 headstage (Axon Instruments, Union City, CA). Macropatches having an average pipette area of 8.3 ± 1 *μ*m^2^ (*N* = 320 patches) (1.6 ± 0.4 MΩ pipette resistance) were used to measure the mean KATP currents and the pharmacological responses of the channels. The mean currents were calculated by subtracting the baseline level from the open-channel level of each current trace and then digitally averaging all generated files by using CLAMPFIT (Axon Instruments, Molecular Devices, Sunnyvale, CA). The baseline level for the KATP current was measured in the presence of internal ATP (5 × 10^−3^ mol/L). Macropatches containing voltage-dependent ion channels or other Kir and showing loss of channel currents during the time of observation were excluded from the analysis. We made no correction for liquid junction potential, which was approximately ±3 mV in our experimental conditions. The concentration–response relationships were constructed by applying increasing concentrations of the drug solution on the internal side of the macropatches excised from FDB, EDL, and SOL muscle fibers. No more than three concentrations of the sulfonylureas and glinides were tested per patch. In our experiments, a period of washout of 25 sec duration was needed to allow a full recovery of the channel currents after the application of the sulfonylureas and glinides solutions to the excised patches.

### Evaluation of the SDH and caspase 3 activities

The SDH activity was measured in the supernatant of the muscle homogenates using the cell Counting Kit-8 (Enzo Life Sciences International, Inc., Farmingdale, NY), which utilizes highly water-soluble tetrazolium salt. WST-8 [2-(2-methoxy-4-nitrophenyl)-3-(4- nitrophenyl)-5-(2,4-disulfophenyl)-2H-tetrazolium, monosodium salt] produces a water-soluble formazan dye upon reduction in the presence of an electron carrier. It is reduced by dehydrogenases to give a yellow-colored product (formazan), which is soluble in the tissue culture medium. The detection sensitivity of CCK-8 is higher than other tetrazolium salts. Succinate dehydrogenase is the component of complex II of the respiratory chain that catalyzes the oxidation of succinate to fumarate in the Krebs cycle. The oxidation of succinate to fumarate is the only Krebs cycle reaction that takes place in the inner membrane itself, as opposed to the other reactions that are catalyzed by soluble enzymes (Kregiel et al. 2008). We expect that the amount of the formazan dye generated by the activity of dehydrogenases in the muscle fibers, particularly that of the inner mitochondrial membrane can be a marker of mitochondrial involvement in the observed atrophy.

The potential role of the KATP channel subunits in the cytotoxic effects of the sulfonylureas and glinides was investigated in HEK293 cells transfected with the KATP channel subunits. The Kir6.2ΔC36, Kir6.2+SUR1 or Kir6.2+SUR2A channel subunits (mouse) were inserted in the mammalian expression vector pcDNA3. The HEK293 cells at 60–80% confluence were transiently cotransfected with 4 *μ*g of DNA that encoded the channel subunits and a lower amount of plasmid DNA that encoded the CD8 receptors using Lipofectamine 2000 and Opti-MEM (Invitrogen, Carlsbad, CA). The cells expressing a specific combination of channel subunits were thereafter incubated for 24 h with the drug solutions under 5% CO_2_–95% O_2_ atmosphere for the maintenance of aerobic conditions, at 37°C. The cell vitality following drugs incubation was evaluated using the Cell-Counting Kit-8 and expressed as% changes of cell viability with respect to the controls.

Caspase-3 activity was measured in the muscle homogenates using a colorimetric assay based on the hydrolysis of the peptide substrate acetyl-Asp-Glu-Val-Asp-*p*-nitroaniline (Ac-DEVD-pNA) by caspase-3 resulting in the release of the pNA which has a high absorbance at 405 nm. The reagents and the CASP-3C kit used were supplied by Sigma Co., Milano, Italy.

### Database searching and data minimization

Database searching was performed using FDA-AERS. AERS is a passive surveillance system that accepts spontaneous reports of adverse events following any US licensed drugs from providers, health care workers, and the public. Although AERS cannot usually prove causal associations between drugs and adverse events and do not give the incidence, it can detect signals to be tested with more rigorous methods including an experimental one. Symptoms recorded on an AERS report were assigned to one or more coding terms using Coding Symbols for Thesaurus of Adverse Reaction Terms. The terms were: muscle atrophy, muscular atrophy, musculoskeletal atrophy, peroneal atrophy, progressive muscular atrophy. This process does not employ standardized case definitions. Reports of hospitalization or prolongation of hospitalization, life-threatening illness, persistent or significant disability/incapacity, or certain other medically important conditions are classified as serious. All other reports are coded as nonserious.

Advanced signal detection and data mining were performed screening AERS reports among the population, we calculated the ratio of the proportion of a particular term among reports following drug administration to the proportion of the same term among serious reports following all other drugs (proportional reporting ratios, PRR) (Evans et al. 2001). We used the proposed criteria for significant disproportionality (coding terms identified in at least three reports, with a PRR > 2.0 and *χ*^2^ > 4.0) to guide selection of AERS reports for further investigation.

To identify positive rechallenge reports (i.e., an adverse event that followed the receipt of a drug and recurred after a subsequent dose), we used the coding term “POS RECHAL” and completed a text search using phrases consistent with a repeat event. Similarly, to identify positive dechallenge reports (i.e., an adverse event that disappeared following dose interruption). Positive derechallenge reports are suggesting a causal association with drugs.

### Data analysis

Data analysis and plot were performed using excel software (Microsoft Office, 2010, Redmond, WA) and SigmaPlot software (Systat Software, Inc., San Jose, CA).

The following four-parameter logistic function was used to fit the concentration–response data:


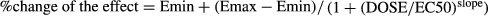


where Emax, is the maximal drug effect, Emin is the minimal drug effect, EC50, is the concentration needed to cause the 50% change in the drug effect, DOSE, is the applied concentration, and slope is the hill slope of the curve.

One-way analysis of variance was used for a multiple comparison of statistically significant differences between drug treatments in the same range of concentrations, and the sample sizes (number of data points) used to calculate the degree of freedom were similar between groups in our experiments. The variance ratio is calculated as: mean square of the drug treatments/mean square of the residuals. This value is then compared with that tabulated at a certain degree of freedom at *P* < 0.05 level of significance. A variance ratio larger than that tabulated indicates a rejection of the null hypothesis and significant drug treatment effects. The absolute efficacy ranking of the drugs were therefore constructed on the basis of the variance analysis. Differences between groups and within groups were evaluated at *P* < 0.05 level of significance. The symbol ≥ was assigned in case of a drug showing a major absolute EC50 and/or Emax values but not statistically significant, while the symbol > was assigned when the calculated EC50 and/or Emax values were statistically significant in respect to those calculated for other drugs. The two-way analysis of variance was used for a multiple comparison of statistically significant differences between drug treatments at different time of incubation.

The percent change in mortality for skeletal muscle fibers was calculated by the following equation: Number of dead cells/Number of total cells in the plate ×100.

The PRR was calculated using the following equation:


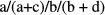


where a is the reaction of interest to a given drug, b is the reaction of interest for all other drugs of the class, c is the all other reactions to a given drug, d all other reactions to all other drugs (Evans et al. 2001). Signal definition: PRR ≥ 2, a minimum of three reports for the reaction of interest, *χ*^2^ ≥ 4. No signal is identified, if PRR is = 1. Duplication of a specific report was evaluated manually and excluded from the analysis. A homemade protocol was used to screen the FDA-AERS database for the association of the identified signal to a specific drug.

The data are expressed as the mean ± SE unless otherwise specified. Significance between pairs of means was calculated by Student's paired *t-*test. Significant differences were considered for *P <* 0.05 or less.

## Results

### Sulfonylureas and glinides induced a muscle type-dependent atrophy in mice

The FDB, SOL, and EDL muscles were incubated for 1, 6, 24, and 48 h with a DMEM+ solution in the absence (controls) or presence of different drugs. We found a significant reduction in the protein content/muscle weight after 24 h of incubation time of FDB muscle with all sulfonylureas (10^−4^ mol/L), glinides (10^−4^ mol/L), and 5HD (500 × 10^−6^ mol/L) with respect to the controls as determined by the two-way analysis of variance. The calculated variance ratio for the sulfonylureas, glinides, 5HD treatments, and controls was higher than that theoretically tabulated at the corresponding degree of freedom indicating that these treatments in FDB muscle were not equal each other and were not affected by the incubation time (*P* < 0.05).

**Figure 1:**
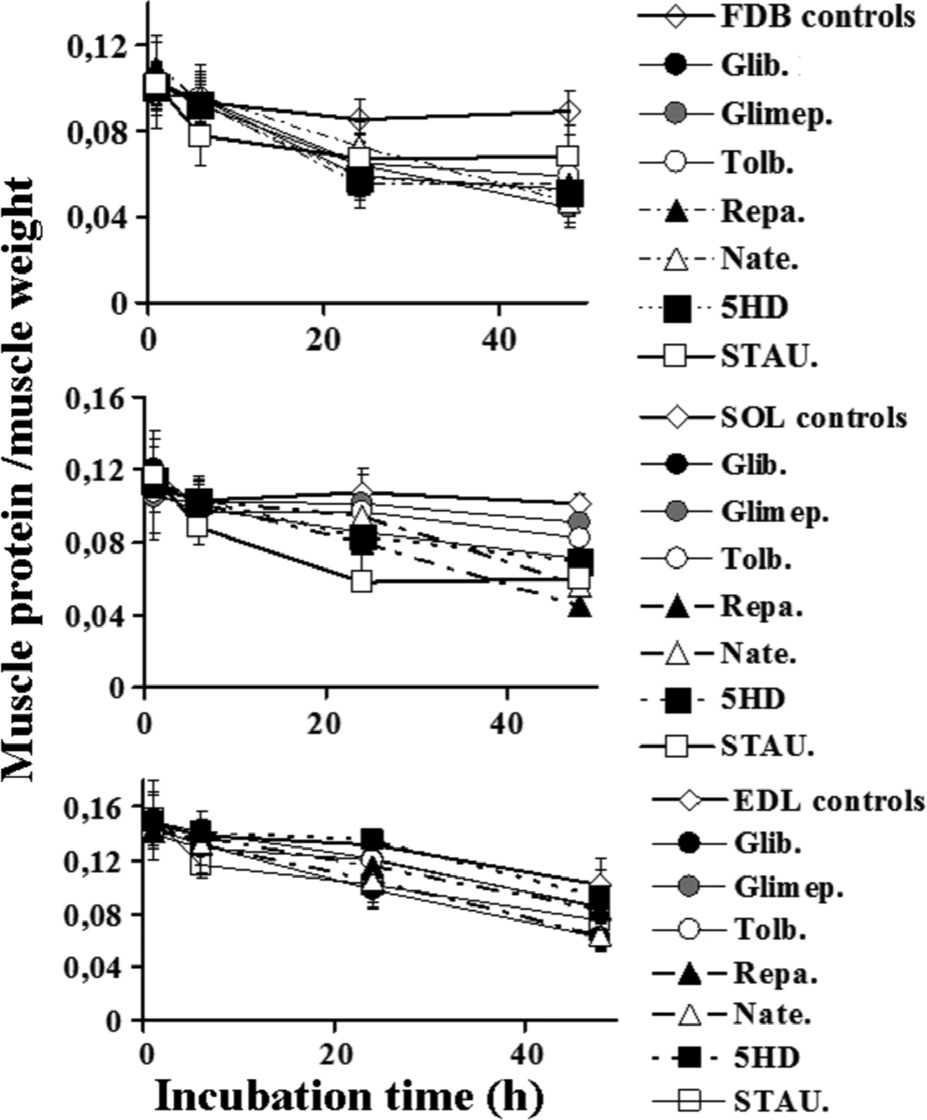
Effects of sulfonylureas, glinides, 5-hydroxydecanoate, and staurosporine on the ratio of muscle protein content/muscle weight of different muscle types of mice at different incubation time. The right hand flexor digitorum brevis (FDB), the extensor digitorum longus (EDL), and the soleus (SOL) muscles were incubated for 1, 6, 24, and 48 h, at 37°C, under 5% CO_2_/95% O_2_ atmosphere, with DMEM+ solution enriched with the glibenclamide (Glib.) (10^−4^ mol/L), glimepiride (Glimep.) (10^−4^ mol/L), tolbutamide (Tolb.) (5 × 10^−4^ mol/L), nateglinide (Nate.) (10^−4^ mol/L), repaglinide (Repa.) (10^−4^ mol/L), 5-hydroxydecanoate (5HD) (5 × 10^−4^ mol/L), and staurosporine (STAU.) (2 × 10^−6^ mol/L). The contralateral left hand muscles (controls) isolated from the same mice were incubated with a DMEM+ solution in the same experimental condition and used as controls. We found a significant reduction in the protein content/muscle weight after 24 h of incubation time of FDB muscle with all drugs under investigation with respect to the controls as determined by the two-way analysis of variance (*P* < 00.5). Repaglinide and glibenclamide were also effective in the SOL and EDL muscles after 24 h of incubation, while glimepiride did not produce a significant reduction in this parameter in these muscle types. The 5HD was effective as atrophic agent in FDB and SOL muscles but it was less effective in EDL. The staurosporine was capable to reduce the protein content in all muscles after 6 h of incubation time. Data are expressed as means±SEM. A minimum of five sample replicates were analyzed for each set of data points.

In SOL muscle, the repaglinide (10^−4^ mol/L), glibenclamide (10^−4^ mol/L), and 5HD (500 × 10^−6^ mol/L) after 24 h of incubation time were capable to reduce significantly the protein content/muscle weight with respect to the controls (Fig. 1). This effect was observed in EDL muscle in the same condition with the repaglinide (10^−4^ mol/L), nateglinide (10^−4^ mol/L), and glibenclamide (10^−4^ mol/L) but not with 5HD.

After 48 h of incubation time, all drugs under investigation were capable to induce a significant reduction in the protein content/muscle weight with respect to the controls in all muscle types.

The apoptotic agent staurosporine (2 × 10^−6^ mol/L) after 6 h of incubation time reduced significantly the protein content/muscle weight in all muscle types (Fig. 1).

**Figure 2:**
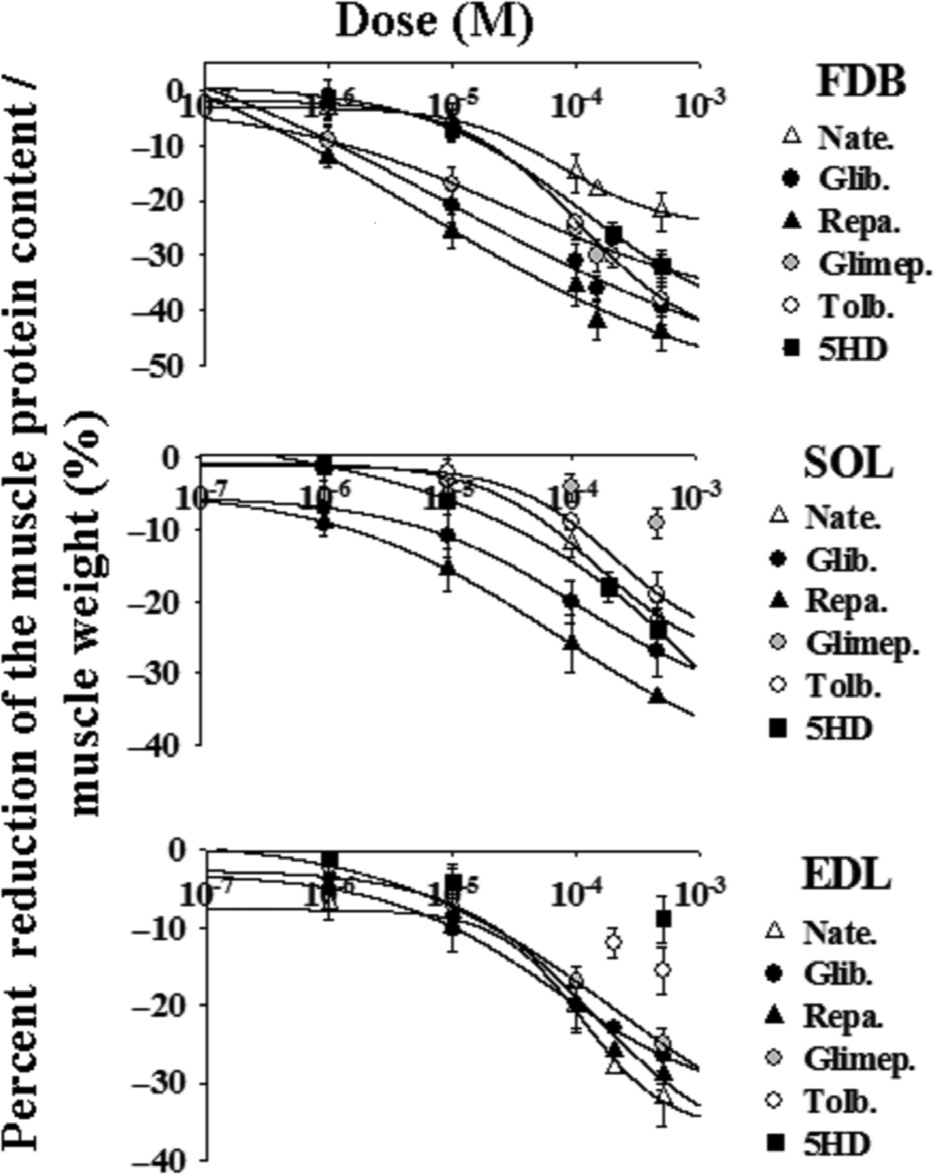
Concentration–response relationships of sulfonylureas, glinides, and 5-hydroxydecanoate concentrations versus the percent reduction in the protein content/muscle weight in all muscle types of mice. These experiments were performed in flexor digitorum brevis (FDB), soleus (SOL), and extensor digitorum longus (EDL) muscles after 24 h of incubation time at 37°C, under 5% CO_2_/95% O_2_ atmosphere, with DMEM+ solution enriched with increasing concentrations (10^−7^ to 5 × 10^−4^ mol/L) of glibenclamide (Glib.), glimepiride (Glimep.), tolbutamide (Tolb.), nateglinide (Nate.), repaglinide (Repa.), and 5-hydroxydecanoate (5HD). These drugs were more effective in FDB and SOL rather than in the EDL muscle. Repaglinide and glibenclamide were the most effective and potent drugs in reducing the protein content in all muscles as determined by the one-way analysis of variance (*P* < 0.05). The data were fitted using a four-parameter logistic Hill function. Data are expressed as means ± SEM. A minimum of five sample replicates were analyzed for each data point.

The incubation of the muscle for 24 h with increasing concentrations of the drugs under investigation concentration dependently reduced the ratio of protein content/muscle weight (Fig. 2). Concentration–response relationship analysis showed that the drugs under investigation were more effective in FDB and SOL as compared with EDL muscle. Repaglinide and glibenclamide were the most effective and potent drugs in reducing the protein content in all muscles (Fig. 2; Table 1). This was confirmed using the one-way analysis of variance that allowed a multiple comparison of different drug treatments performed in the same range of concentrations. This analysis showed that the calculated variance ratio for the repaglinide and glibenclamide treatments was higher than that theoretically tabulated at the corresponding degree of freedom indicating that these drug treatments in FDB and SOL muscles were not equal to that of the other drugs (*P* < 0.05). The order of efficacy of the sulfonylureas and glinides in reducing the protein content in FDB muscle based on the one-way analysis of variance was repa.≥glib.>glimep.>tolb.>nate. and it was repa.≥glib.>nate.≥tolb.>glimep. in SOL muscle (Table 1). While, the order of efficacy of the drugs in reducing the protein content in EDL muscle was glib.≥repa.≥nate.>glimep.>tolb. (Table 1). The 5HD was more potent and effective as an atrophic agent in the SOL and FDB muscles than in EDL muscle.

**Table 1:** Fitting parameters of concentration–response relationships of the sulfonylureas and glinides concentrations versus the percent changes of the protein content/muscle weight in different muscles

Treatment	Emax (%)	Emin (%)	EC50 (10^−6^ mol/L)	Slope factor *n*
Glibenclamide				
FDB	−56.36 ± 9^1^	−5.98 ± 0.1	8.84 ± 0.1^1^	0.29 ± 0.01
EDL	−33.12 ± 6	−2.91 ± 0.2	66.6 ± 6	0.62 ± 0.02
SOL	−35.52 ± 7^1^	−5.43 ± 0.3	91.1 ± 4^1^	0.61 ± 0.03
Tolbutamide				
FDB	−47.08 ± 9	−1.59 ± 0.1	107.1 ± 11	0.89 ± 0.01
EDL	−18.8 ± 7	–	–	–
SOL	−26.56 ± 6	−1.20 ± 0.2	219.2 ± 12	1.03 ± 0.01
Glimepiride				
FDB	−40.42 ± 8	−2.39 ± 0.1	29.3 ± 9	0.45 ± 0.01
EDL	−28.47 ± 7	−1.44 ± 0.2	202.1 ± 12	0.45 ± 0.02
SOL	−10.1 ± 6	–	–	–
Repaglinide				
FDB	−57.52 ± 9^1^	−1.39 ± 0.1	5.21 ± 2^1^	0.32 ± 0.01
EDL	−39.95 ± 4	−2.62 ± 0.2	139.1 ± 17	0.75 ± 0.02
SOL	−45.72 ± 6^1^	−4.30 ± 0.3	71.5 ± 7^1^	0.46 ± 0.02
Nateglinide				
FDB	−24.73 ± 2	−2.94 ± 0.1	161.2 ± 12	1.05 ± 0.01
EDL	−35.99 ± 3	−7.71 ± 0.3	110.1 ± 12	1.27 ± 0.02
SOL	−29.52 ± 4	−0.73 ± 0.1	180.1 ± 11	0.92 ± 0.01
5-hydroxydecanoate				
FDB	−44.81 ± 9	−0.63 ± 0.1	113.3 ± 12	0.63 ± 0.03
EDL	−5.31 ± 1	–	–	–
SOL	−38.81 ± 2	−0.63 ± 0.1	151.5 ± 14	0.62 ± 0.03

The muscles were incubated for 24 h in the presence of increasing concentrations of the drugs. The data are the mean ± SE of the maximal drug effect, Emax; the minimal drug effect, Emin; the concentration needed to cause the 50% change in the drug effect, EC50; the hill slope of the curve, *n*. Treatment indicates the drug treatment in different muscles. FDB, flexor digitorum brevis; SOL, soleus; EDL, extensor digitorum longus.

1 Data significantly different between treatments evaluated by the one-way analysis of variance in different muscles (*P* < 0.05).

### Effects of the glibenclamide and repaglinide on fiber viability

**Figure 3:**
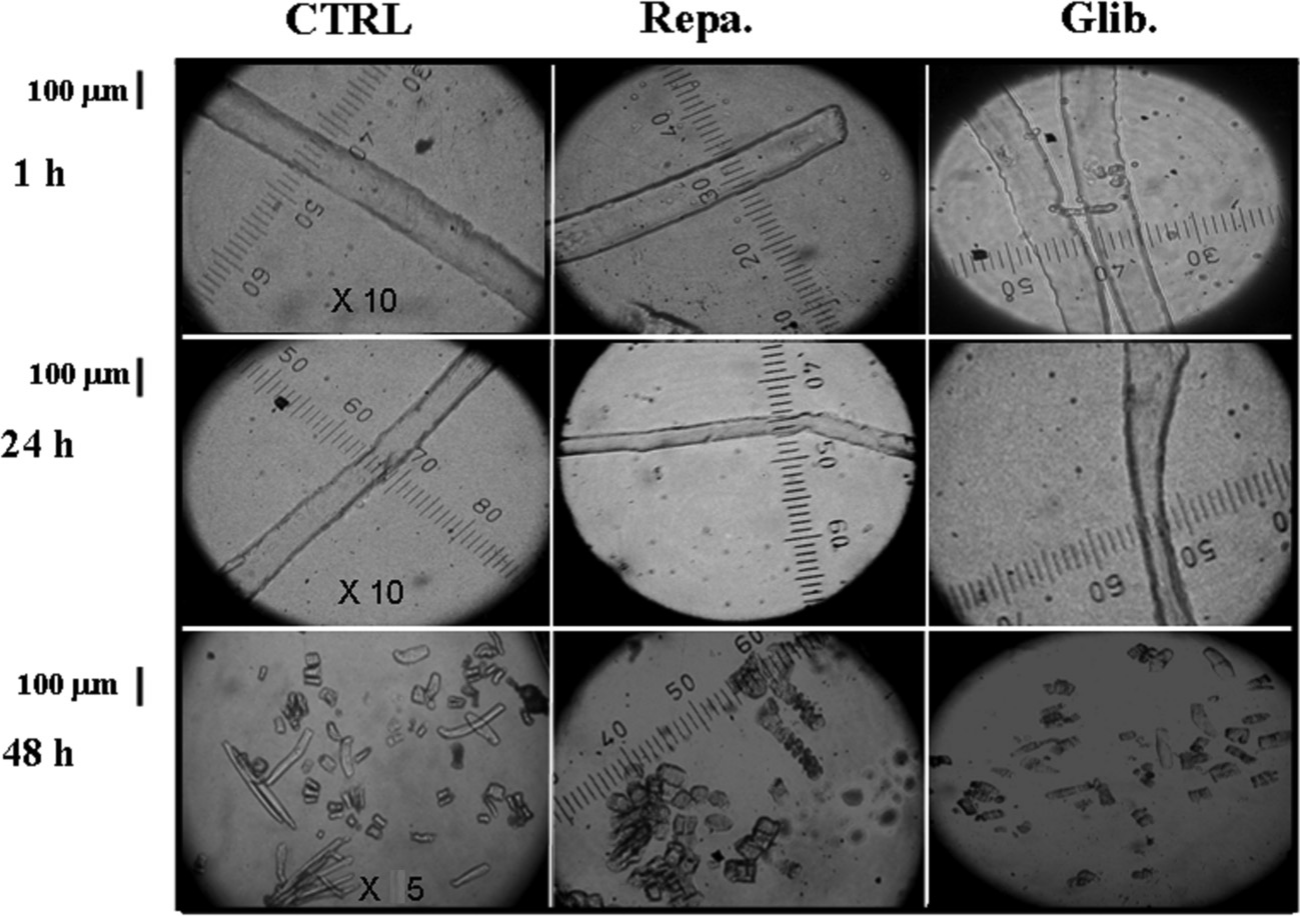
Images of enzymatically isolated fibers from flexor digitorum brevis (FDB) muscles after treatment with the glibenclamide (Glib.) and repaglinide (Repa.) at different incubation time. The fiber diameter and morphology were measured at a 10× magnification; mortality was evaluated at a 5× magnification using QuantiCell 900 integrated imaging system. Isolated fibers, before analysis, were equilibrated in DMEM (300 mOsmol L) for 15 min at 25°C. Fiber diameter and mortality following incubation for 1–48 h with DMEM solution used as control and DMEM solution + glibenclamide (10^−5^ mol/L) and repaglinide (10^−5^ mol/L). No effects were observed after 1 h incubation time in the presence of the drugs. The glibenclamide and repaglinide treatments reduced the fiber diameter after 24 h of incubation with respect to the controls. The fiber mortality increased with drug treatments with respect to the control after 48 h of incubation time.

The “in vitro” effects of the glibenclamide and repaglinide (10^−5^ mol/L) were investigated on FDB fibers morphology and viability. The isolated FDB fibers were incubated for 1, 24, and 48 h with DMEM+ solution used as controls or DMEM+ solution enriched with the drugs. The incubation of the fibers for 24 h in the presence of 10^−5^ mol/L concentrations of glibenclamide and repaglinide solutions significantly reduced the diameter with respect to the controls (Table 2; Fig. 3). The drug treatments increased fiber mortality evaluated by monitoring the morphological parameters of the fibers such as the length and diameters or the surface blebs appearance as well as their contractility response to ATP (1 mmol/L) and/or KCl (150 mmol/L).

**Table 2:** “In vitro” effects of the glibenclamide, repaglinide, and diazoxide on flexor digitorum brevis muscle FDB muscle fiber viability in mice

		Time of Incubation (h)		
		
Treatment	Parameters	1	24	48
Controls	Diam. (*μ*m)	87.2 ± 2.7 (Nf = 35)	83.7 ± 2.5 (Nf = 31)	85.2 ± 2.5 (Nf = 28)
	Mort. (%)	1 ± 0.11 (Npl = 5)	36 ± 8 (Npl = 5)	68 ± 10 (Npl = 5)
Glibenclamide (10^−5^ mol/L)	Diam. (*μ*m)	84 ± 3 (Nf = 28)	72 ± 3.3^1^ (Nf = 28)	69 ± 6.1^1^ (Nf = 22)
	Mort. (%)	8 ± 0.9 (Npl = 5)	55 ± 8^1^ (Npl = 5)	98 ± 9^1^ (Npl = 5)
Repaglinide (10^−5^ mol/L)	Diam. (*μ*m)	83.8 ± 2.4 (Nf = 25)	76.5 ± 2.4^1^ (Nf = 28)	70.1 ± 4.1^1^ (Nf = 19)
	Mort. (%)	7.3 ± 3 (Npl = 5)	52 ± 8^1^ (Npl = 5)	99 ± 6^1^ (Npl = 5)
Diazoxide (250 × 10^−6^ mol/L)	Diam. (*μ*m)	83 ± 6 (Nf = 15)	82 ± 7.2 (Nf = 24)	80.7 ± 3 (Nf= 28)
	Mort. (%)	1 ± 0.3 (Npl = 5)	28 ± 7 (Npl = 5)	58 ± 8 (Npl = 5)
Glibenclamide (10^−5^ mol/L) + Diazoxide (250 × 10^−6^ mol/L)	Diam. (*μ*m)	81 ± 4 (Nf = 20)	79 ± 3.1 (Nf = 23)	85.7 ± 3^2^ (Nf = 28)
	Mort. (%)	1.4 ± 0.4^2^ (Npl = 5)	38 ± 9^2^ (Npl = 5)	59 ± 8^2^ (Npl = 5)
Repaglinide (10^−5^ mol/L) + Diazoxide (250 × 10^−6^ mol/L)	Diam. (*μ*m)	82 ± 5.1 (Nf = 21)	83 ± 4.1 (Nf = 25)	85.9 ± 5.1^2^ (Nf = 26)
	Mort. (%)	1.5 ± 0.6^2^ (Npl = 5)	40 ± 8^2^ (Npl = 5)	58 ± 10^2^ (Npl = 5)
Staurosporine (2 × 10^−6^ mol/L)	Diam. (*μ*m)	78.6 ± 4.4 (Nf = 29)	66.5 ± 8.4^1^ (Nf = 29)	54.5 ± 9.4^1^ (Nf = 29)
	Mort. (%)	16.3 ± 6 (Npl = 5)	78 ± 9^1^ (Npl = 5)	96.2 ± 11^1^ (Npl = 5)

The data are the mean ± SE of the diameter, Diam.; mortality, Mort. Treatment indicates the treatment in the presence of drugs or in the absence of drugs, controls. Nf, number of fibers; Npl, number of analyzed plates. The % of fibers mortality were fibers showing more than 40% change in the morphological parameters (length and diameters, blebs) and fails contractile response to ATP (1 mmol/L) or KCl (15 mmol/L).

1 Data significantly different between treatments evaluated by the two ways analysis of variance (*P* < 0.05).

2 Data significantly different between treatments (repaglinide-diazoxide and glibenclamide-diazoxide data vs. repaglinide and glibenclamide data) evaluated by the two ways analysis of variance (*P* < 0.05).

The long-term incubation (48 h) of the fibers increased the cellular mortality with respect to the controls with all drugs under investigation and this effect was associated with the loss of the fibers contractility in response to ATP (1 mmol/L) and/or high external K^+^ ions (150 mmol/L) concentrations (Table 2).

The staurosporine (2 × 10^−6^ mol/L) was more effective than glibenclamide and repaglinide in inducing changes of the fiber morphology and in increasing the fibers mortality.

### Effects of diazoxide on muscle protein content and cell viability in mice

**Figure 4:**
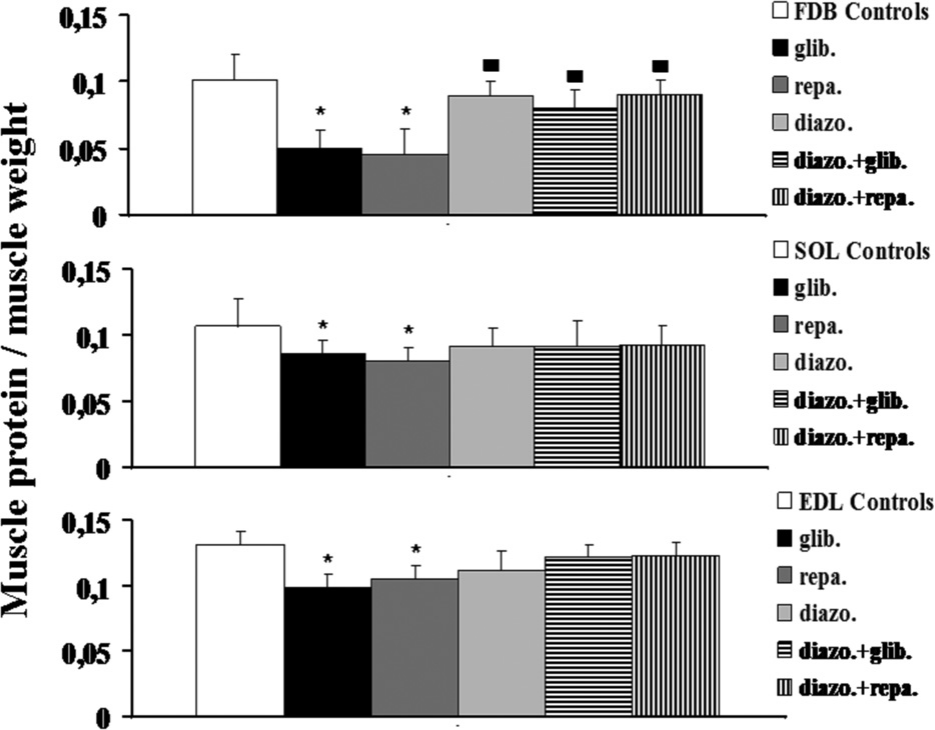
Cytoprotective effects of diazoxide against the muscle atrophy induced by glibenclamide and repaglinide in mice. The right hand flexor digitorum brevis (FDB), the extensor digitorum longus (EDL), and the soleus (SOL) muscles were incubated for 24 h, at 37°C, under 5% CO_2_/95% O_2_ atmosphere, with DMEM+ solution enriched with the diazoxide (Diazo.) (250 × 10^−6^ mol/L), glibenclamide (Glib.) (10^−4^ mol/L), repaglinide (Repa.) (10^−4^ mol/L), and the combination of glibenclamide or repaglinide (10^−4^ mol/L) and diazoxide (250 × 10^−6^ mol/L). The contralateral left hand muscles (controls) isolated from the same mice were incubated with a DMEM+ solution in the same experimental condition and used as controls. Diazoxide treatment, per se, did not affect the protein content in all muscles. The coincubation of the muscles with diazoxide + glibenclamide or repaglinide prevented the atrophy induced by these drugs in all muscles. The diazoxide-repaglinide or diazoxide-glibenclamide data were significantly different in respect to the glibenclamide or repaglinide data in FDB muscle. No significant differences were observed between the diazoxide-repaglinide or diazoxide-glibenclamide data and controls. The data are expressed as means ± SEM of a minimum of five sample replicates. The data (*) were significantly different with respect to the controls and (▪) to the glibenclamide or repaglinide data (*P* < 0.05) as determined by student *t*-test.

Diazoxide (250 × 10^−6^ mol/L) after 24 h of incubation time did not affect the protein content in all muscle types (Fig. 4). This drug was capable to fully prevent the reduction in the muscle protein content induced by repaglinide and glibenclamide in the FDB, SOL, and EDL muscles. No significant differences were found between the experimental groups and controls as evaluated by the one-way analysis of variance (*P* < 0.05).

Diazoxide prevented the reduction in the fiber diameter and the cellular death induced by glibenclamide and repaglinide (Table 2).

### Effects of KATP channel blockers on skeletal muscle KATP channel currents in mice

**Figure 5:**
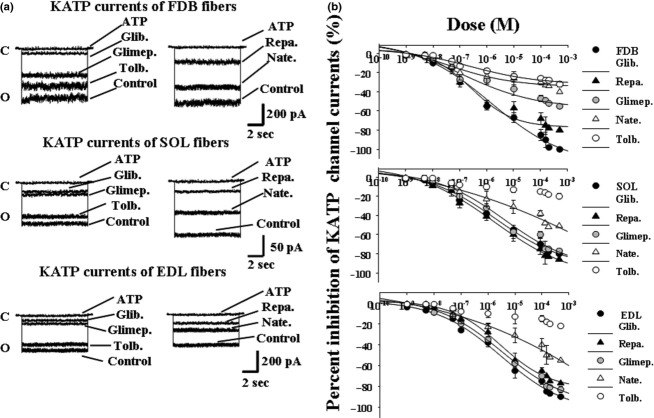
Effects of the sulfonylurea and glinides on ATP-sensitive K^+^ channel (KATP) current in skeletal muscle fibers of mice. (A) Sample traces of KATP channel currents recorded at −60 mV (Vm) in excised macropatches from flexor digitorum brevis (FDB), soleus (SOL), and extensor digitorum longus (EDL) muscle fibers in the presence of 150 mmol/L KCl on both sides of the membrane patches, at 25°C. The drug solutions (10^−4^ mol/L) were applied on the internal side of the membrane patches. The glibenclamide (Glib.), glimepiride (Glimep.), tolbutamide (Tolb.), nateglinide (Nate.), and repaglinide (Repa.) reduced the KATP channel currents with different efficacy. C, closed channel level; O, open-channel level. (B) Concentration–response relationship of the percent reduction in the KATP channel current versus drug concentrations was performed on currents recorded, at −60 mV (Vm), in excised macropatches in the presence of 150 mmol/L KCl on both sides of the membrane patches, at 25°C, in the FDB, SOL, and EDL muscle fibers. Increasing concentrations (10^−9^ to 5 × 10^−4^ mol/L) of the drug solutions were applied to the internal sides of the membrane patch. The most potent and effective drugs in reducing the KATP channel current were repaglinide and glibenclamide in all muscle fibers. Nateglinide and tolbutamide were the less potent and effective drugs in all fibers. The data were fitted using a four-parameter logistic Hill function. Data are expressed as means ± SEM of a minimum of four macropatches.

The “in vitro” effects of sulfonylureas and glinides on the sarcolemma KATP channel in isolated skeletal muscle fibers were investigated in excised-patch experiments. The channel current amplitude recorded in mouse skeletal muscle, in the presence of 150 mmol/L concentration of KCl on both sides of the membrane patches, at −60 mV (Vm), was higher in FDB than in EDL and SOL muscle fibers as observed in rat (Tricarico et al. 2006). The mean current amplitude in the FDB, EDL, and SOL fibers was −390.9 ± 12 pA (Number of patches = 49), −281.5 ± 21 pA (Number of patches = 52), and −130.5 ± 9 pA (Number of patches = 33), respectively. The application of the glinides and sulfonylureas to the internal side of the patches inhibited the KATP channel currents (Fig. 5A). Concentration–response relationship analysis showed that the drugs under investigation were more effective in FDB and SOL than in EDL fibers (Fig. 5B). Repaglinide and glibenclamide were the most effective drugs as KATP channel blockers in all muscles. The data were fitted with one inhibitory site function in all muscles (Table 3). The order of efficacy of the sulfonylureas and glinides in blocking the sarcolemma KATP channel based on the one-way analysis of variance was glib.≥repa.>glimep.>nate≥tolb. in FDB fibers which is similar to that observed as atrophic agents (Table 3). In SOL and EDL fibers, the order of efficacy of these drugs as KATP channel blockers were repa. ≥glib.≥glimep.>nate.>tolb. and glib.≥glimep.≥repa.>nate>tolb., respectively, which were not related with their capability to induce atrophy. These findings suggest that the sarcolemma KATP channel plays a major role in the sulfonylureas and glinides-induced atrophy in FDB but not in SOL or EDL muscles.

**Table 3:** Fitting parameters of concentration–response relationships of the sulfonylureas and glinides concentrations versus the percent changes of the KATP channel current in different muscle phenotypes

Treatment	Emax (%)	Emin (%)	EC50 (10^−6^ mol/L)	Slope factor *n*
Glibenclamide				
FDB	−98.9 ± 9^1^	−2.4 ± 1	1.08 ± 0.2^1^	0.91 ± 0.01
EDL	−78.1 ± 6^1^	−2.9 ± 1	30.7 ± 7^1^	0.82 ± 0.02
SOL	−70.5 ± 4^1^	−3.2 ± 0.7	35.1 ± 2^1^	0.75 ± 0.03
Tolbutamide				
FDB	−40.6 ± 8	−1.59 ± 0.2	170.1 ± 11	0.61 ± 0.04
EDL	−26.3 ± 5	–	–	–
SOL	−19.1 ± 3	–	–	–
Glimepiride				
FDB	−45.4 ± 9	−2.3 ± 0.1	36.7 ± 10	0.93 ± 0.03
EDL	−80.4 ± 7^1^	−1.9 ± 0.2	48.8 ± 8^1^	0.91 ± 0.02
SOL	−75.3 ± 6^1^	−2.1 ± 0.3	55.1 ± 11^1^	0.90 ± 0.05
Repaglinide				
FDB	−75.4 ± 9	−1.3 ± 0.2	1.67 ± 0.9^1^	0.93 ± 0.04
EDL	−70.4 ± 10	−4.9 ± 0.5	55.6 ± 11^1^	0.82 ± 0.07
SOL	−78.3 ± 8	−3.1 ± 0.5	17.11 ± 4^1^	0.90 ± 0.08
Nateglinide				
FDB	−48.7 ± 7	−1.3 ± 0.4	132.1 ± 18	0.78 ± 0.07
EDL	−50.3 ± 6	−4.9 ± 0.5	120.1 ± 11	0.67 ± 0.09
SOL	−55.2 ± 9	−3.1 ± 1	110.1 ± 21	0.71 ± 0.08

The data are the mean ± SE of the maximal drug effect, Emax; the minimal drug effect, Emin; the concentration needed to cause the 50% change in the drug effect, EC50; the hill slope of the curve, *n*. Treatment indicates the drug treatments in different muscles. FDB, flexor digitorum brevis; SOL, soleus; EDL, extensor digitorum longus.

1 Data significantly different evaluated by the one-way analysis of variance in different muscles (*P* < 0.05).

### Effects of the KATP channel modulators on SDH and caspase 3-activities in skeletal muscle of mice

**Figure 6:**
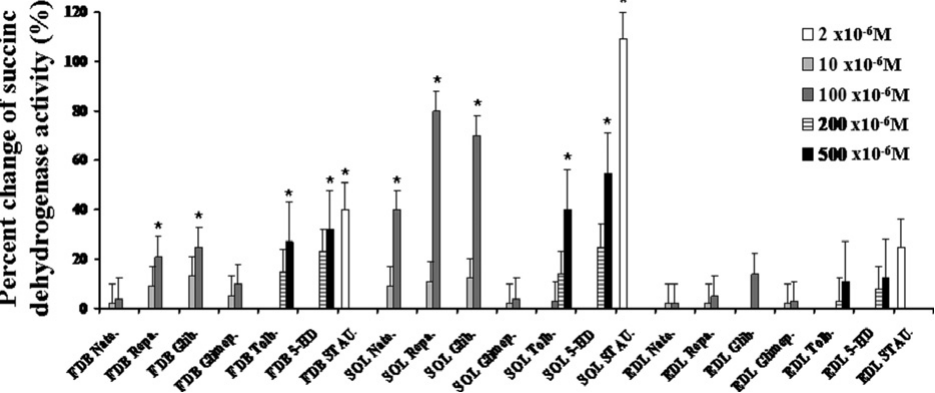
Effects of the sulfonylureas, glinides, and 5-hydroxydecanoate on dehydrogenase activity of muscles in mice. The dehydrogenase activity was expressed as the percent changes of the enzyme activity with respect to that measured in the contralateral controls. The dehydrogenase activity was measured in the flexor digitorum brevis (FDB), extensor digitorum longus (EDL), and soleus (SOL) muscles incubated for 24 h with 5-hydroxydecanoato (5-HD) (200–500 × 10^−6^ mol/L), glibenclamide (Glib.) (10^−5^ to 10^−4^ mol/L), tolbutamide (Tolb.) (100–500 × 10^−6^ mol/L), and glimepiride (Glimep.) (10^−5^ to 10^−4^ mol/L), nateglinide (Nate.) (10^−5^ to 10^−4^ mol/L), repaglinide (Repa.) (10^−5^ to 10^−4^ mol/L). All drugs were capable to significantly enhance the dehydrogenase activity in the SOL muscle with respect to the controls but not glimepiride. Glibenclamide, repaglinide, tolbutamide, and 5HD caused a mild and significant increase in the dehydrogenase activity also in the FDB muscle. All drugs did not affect the dehydrogenase activity in the EDL muscle. The apoptotic agent staurosporine (STAU.) (2 × 10^−6^ mol/L) significantly enhanced this parameter in all muscle types. The data (*) were significantly different with respect to the controls (*P* < 0.05) as determined by student *t*-test.

The incubation of SOL muscle for 24 h in the presence of glibenclamide (10^−4^ mol/L), tolbutamide (500 × 10^−6^ mol/L), repaglinide (10^−4^ mol/L), nateglinide (10^−4^ mol/L), and 5-HD (200–500 × 10^−6^ mol/L) solutions significantly enhanced the SDH activity with respect to the contralateral controls (Fig. 6). While, glimepiride (10^−4^ mol/L) failed to enhance the dehydrogenases activity in this muscle. Staurosporine (2 × 10^−6^ mol/L) significantly enhanced the dehydrogenases activity in SOL but not in FDB and EDL muscles. The order of efficacy of these drugs in enhancing the SDH activity based on the one-way analysis of variance was repa.≥glib.>nate.≥tolb.>glimep. which is similar to that observed as atrophic agents in SOL muscle. A significant enhancement of SDH was observed in the FDB muscle in the presence of repaglinide, glibenclamide, tolbutamide, and 5HD which was, however, less than that observed in SOL muscle (Fig. 6). The order of efficacy of these drugs in enhancing the SDH activity based on the one-way analysis of variance in FDB muscle was tolb.> glib.≥repa.>nate. No significant change in this parameter was observed in the EDL muscle with the drugs under investigation. The 5HD was more effective in enhancing the SDH activity in the SOL and FDB muscles than in EDL muscle.

Repaglinide (10^−4^ mol/L), glibenclamide (10^−4^ mol/L) (*n* = 5), 5-HD (500 × 10^−6^ mol/L) (*n* = 5), nateglinide (10^−4^ mol/L) (*n* = 5), and tolbutamide (500 × 10^−6^ mol/L) (*n* = 5) significantly enhanced caspase-3 activity in SOL muscle after 24 h of incubation time from 0.987 ± 0.06 (10^−6^ *μ*mol pNA·min^−1^·mL^−1^) in the contralateral controls (*n* = 9) to 1.651 ± 0.08 (*n* = 5) (*P* < 0.05), 1.613 ± 0.06 (*n* = 5) (*P* < 0.05), 1.16 ± 0.09 (*n* = 5) (*P* < 0.05), 1.24 ± 0.03 (*n* = 5) (*P* < 0.05) and 1.22 ± 0.03 (*n* = 5) (*P* < 0.05), respectively, in the treated muscles as evaluated by student *t*-test. In contrast, glimepiride (10^−4^ mol/L) did not affect caspase-3 activity in this muscle type. Staurosporine significantly enhanced caspase-3 activity in SOL muscle from 0.921 ± 0.05 (*n* = 4) in the contralateral controls to 2.834 ± 0.05 (*n* = 6) (*P* < 0.05) in the treated muscles but not in FDB and EDL muscles.

Not a significant enhancement of caspase-3 activity was measured in the EDL and FDB muscles treated with all drugs under investigation.

These findings suggest that repaglinide, glibenclamide, tolbutamide, nateglinide, and 5-HD induced atrophy in SOL muscle mostly activating a mitochondrial pathway possibly associated with mito-KATP channel and enhancing the caspase-3 activity.

### Regulation of the cell viability by sulfonylureas and glinides in HEK293 cells that were expressing the KATP channel subunits

**Figure 7:**
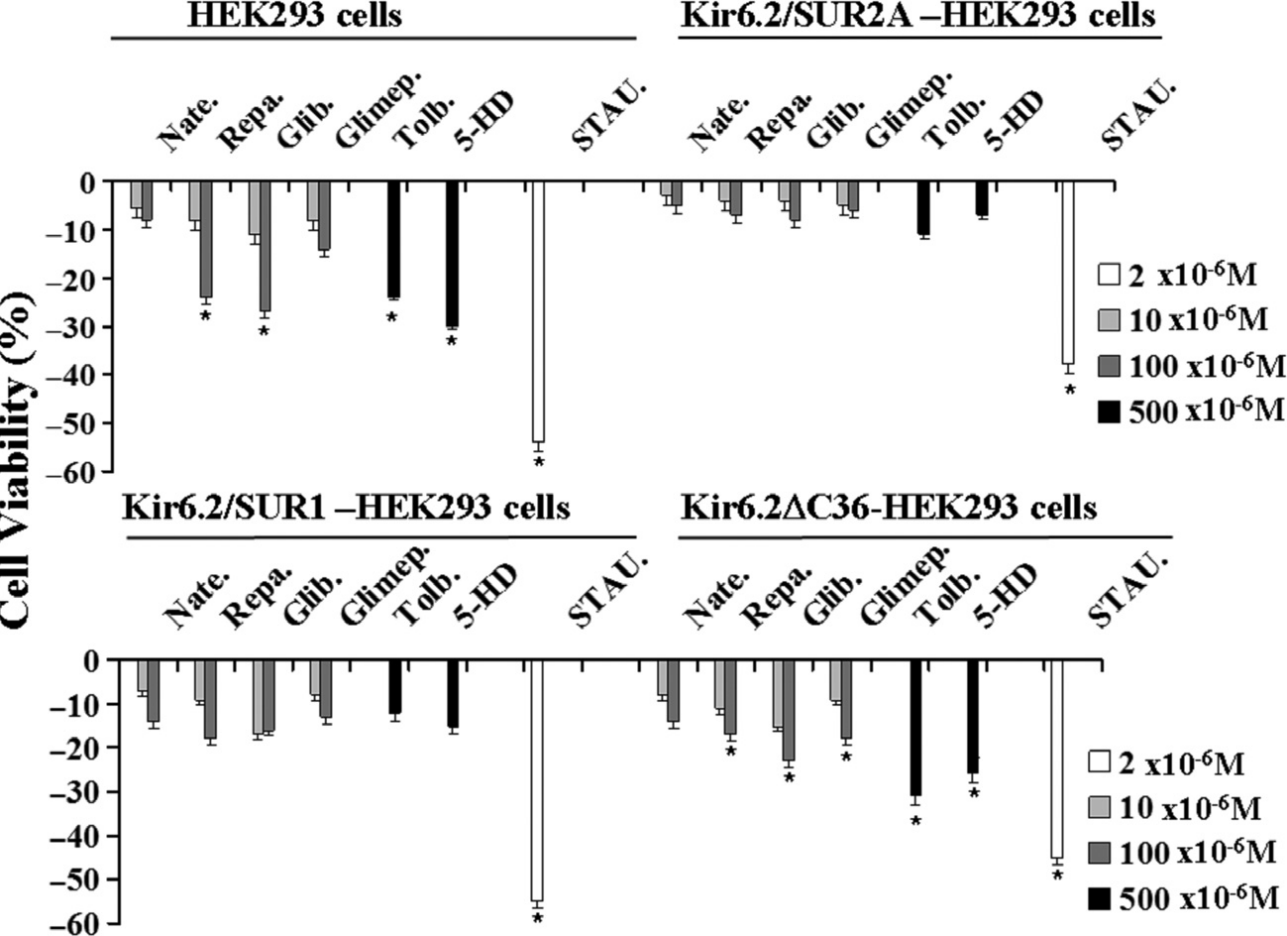
Effects of the sulfonylureas, glinides, 5-hydroxydecanoate, and staurosporine on cell viability in HEK293 cells transfected with the KATP channel subunits. The drug effects on cell viability were investigated in not transfected HEK293 cells or in cells that were expressing the Kir6.2ΔC36, Kir6.2+SUR1, and Kir6.2+SUR2A channel subunits. The cells were incubated for 24 h in the presence of glibenclamide (Glib.), glimepiride (Glimep.), repaglinide (Repa.), nateglinide (Nate.) (10–100 × 10^−6^ mol/L), tolbutamide (Tolb.) (500 × 10^−6^ mol/L), 5-hydroxydecanoate (5-HD) (500 × 10^−6^ mol/L), and staurosporine (STAU.) (2 × 10^−6^ mol/L) solutions under 5% CO_2_–95% O_2_ atmosphere for the maintenance of aerobic conditions, at 37°C. The cell viability was evaluated in the presence of the drugs using the Cell-Counting Kit-8 and expressed as% changes of cell viability with respect to the controls. The glibenclamide and repaglinide exerted a mild cytotoxicity in cells transfected with SUR1-KIR6.2 leading to a reduction in the cell viability of about 18–20% in our experimental condition. In contrast, no effects of the drugs were observed in the cell line expressing the SUR2A-Kir6.2 subunits. The observed effects of the drugs in the not transfected cells and in Kir6.2ΔC36-HEK293 cells in the absence of SUR subunits can be related with off-target effects. The data (*) were significantly different with respect to the controls (*P* < 0.05) as determined by student *t*-test.

The effects of the glibenclamide (10–100 × 10^−6^ mol/L), tolbutamide (500 × 10^−6^ mol/L), glimepiride (10–100 × 10^−6^ mol/L), glinides (10–100 × 10^−6^ mol/L), and 5-HD (500 × 10^−6^ mol/L) on cell viability were investigated in HEK293 cells transfected with the cDNA plasmid encoding for Kir6.2+SUR1, Kir6.2+SUR2A subunits or Kir6.2ΔC36 subunit alone (Fig. 7). We showed that glibenclamide and repaglinide exerted a mild cytotoxicity in cells transfected with SUR1-KIR6.2 leading to a reduction in the cell viability of about 18–20% in our experimental condition. In contrast, no effects of the drugs were observed in the cell line expressing the SUR2A-Kir6.2 subunits. The observed effects of the drugs in the not transfected cells and in Kir6.2ΔC36-HEK293 cells in the absence of SUR subunits can be related with off-target effects as glibenclamide blocks Kir6.2ΔC36 channel to a considerable amount as well (Gribble et al. 1998). It is impossible to compare results from cells transfected with Kir6.2ΔC36 with those from cells transfected with complete KATP channels as the quantitative expression of the delta-channel is very poor. In line with this observation, the data shown for Kir6.2ΔC36-HEK293 cells are very similar to untransfected cells. Our findings are in line with the report of Hambrock et al. (2006), who showed that glibenclamide induced cell detachment and apoptosis in cell line transfected with SUR1 subunit, while no effects were reported by these authors in cell line transfected with SUR2B subunit (SUR2A was not tested) (Hambrock et al. 2006). Some effects of glibenclamide were observed in cell line not expressing channel subunits but transfected only with the vector (Hambrock et al. 2006).

In contrast, the apoptotic agent staurosporine (2 × 10^−6^ mol/L) significantly reduced the cell viability in all cell lines.

### Muscle atrophy signal associated with glyburide/glibenclamide treatment in humans

From 6 October 2011, through 29 June 2012, FDA-AERS received 1.697.582 reports of adverse events. Among these, muscle atrophy as a coding term was reported in 0.022% of the total reports for all drugs not related with sulfonylureas or glinides, while the atrophic reactions identified by others related terms such as muscular atrophy, musculoskeletal atrophy, peroneal atrophy, and progressive muscular atrophy were not found in the analyzed reports. Muscle atrophy was reported in 0.27% of the glibenclamide/glyburide reports, while it was not found in the tolbutamide, glimepiride, repaglinide, and nateglinide reports in the same period of observation. The data mining analysis performed by calculating the PRR revealed a significant association of the muscle atrophic events with the glyburide (Table 4). No positive rechallenge or dechallenge data were found in the analyzed case reports. Muscle atrophy was reported in 0.27% of the dexamethasone reports. The number of muscle atrophy signals for dexamethasone, which is known to induce atrophy in human, were 20 with a calculated PRR of 12.2 (Chi Q = 9.2). In five cases, the drug was considered the principal suspected drug responsible for the atrophy observed, in nine cases was considered the secondary suspected drug and in the six cases was considered as a concomitant drug.

**Table 4:** Number of adverse events reported in the FDA-AERS database and proportional reporting ratio

ADRs (8 months) October 2011–June 2012	Glyburide/Glibenclamide	Glimepiride	Tolbutamide	Repaglinide	Nateglinide	All drugs
Muscle atrophy (*N*)	4 c (PRR = 12) (Chi Q = 0.3.)	0	0	0	0	373
Total (*N*)	1460	945	34	337	83	1,697,582

ADR, adverse drug reactions; PRR, proportional reporting ratio; c, indicates concomitant drug treatment.

However, it remains unexplained why the equipotent compounds in “in vitro” experiments as atrophic agents and KATP channel blockers such as glibenclamide and repaglinide fail to result in the same number of adverse reports in humans. To try to solve this question, we investigated hypoglycemia reports for the compounds investigated as a measure of potency and see if that correlates with the reports of atrophy. We found that glybenclamide, in a period of observation of 3 months, leads to 37 cases of severe hypoglycemia (4.8%); in the same period nine and six cases of hypoglycemia reports for glimepiride (1.1%) and repaglinide (0.78%), respectively, were found. No cases were reported for nateglinide and tolbutamide. A total of 767 cases of hypoglycemia reports were collected in the same period of observation for all drugs unrelated with sulfonylureas and glinides.

## Discussion

Pharmacological investigation showed that the sulfonylureas and glinides investigated here caused a muscle type-dependent atrophy in mice. The rank order of the atrophic effects observed in different muscle types with these drugs was: FDB>SOL>EDL. The enhanced response of the FDB muscle to the atrophic action of the sulfonylureas and glinides can be explained by the fact that the FDB muscle fibers are characterized by an elevated sarcolemma KATP channel current as compared with SOL and EDL muscles as observed in our experiments. The sulfonylureas and glinides exert atrophic effect in FDB muscle interacting with the sarcolemma KATP channel. This is demonstrated by the fact that the order of efficacy of the sulfonylureas and glinides in blocking the sarcolemma KATP channel based on the one-way analysis of variance is glib.≥repa.>glimep.> nate≥tolb. which is similar to that observed as atrophic agents in the FDB muscle being repa.≥glib.> glimep.>tolb.≥nate. Repaglinide, glibenclamide, and glimepiride are known to inhibit cloned SUR1-type at nanomolar concentrations and SUR2-type channels at micromolar concentrations and were more potent and effective as channel blockers and atrophic agents in FDB fibers expressing either subunits (Gribble and Reimann 2003). As previously shown in rat, the FDB fibers in mice are characterized by enhanced KATP channel currents possibly sustained by Kir6.2/SUR2A and Kir6.2/SUR1 subunits with respect to EDL and SOL muscles (Tricarico et al. 2006, 2010). Tolbutamide and nateglinide are known to block preferentially the SUR1-type channel and were the less effective drugs in the SOL and EDL muscles expressing preferentially the SUR2A subunit (Tricarico et al. 2006). This suggests that the block of the sarco-KATP channel plays a major role in determining the observed atrophy in this muscle type but a minor role in the SOL and EDL muscles.

Furthermore, repaglinide, glibenclamide, and tolbutamide caused an enhancement of the SDH activities in the atrophic FDB muscles suggesting a possible involvement of a mitochondrial target in their actions. The FDB muscle is indeed composed of fast-twitch glycolytic/oxidative fibers characterized by an elevated mitochondrial density as compared with that reported in the EDL muscle suggesting that the interaction of the sulfonylureas and glinides with a mito-KATP channel may be an additional mechanism operative in this muscle type.

Despite of the significant atrophy induced by glimepiride in the FDB fibers, this drug is a potent sarcolemma KATP channel blocker in our experiments, caused a mild cytotoxicity in HEK293 cell line expressing the surface SUR1 subunit but did not affect mito-dehydrogenases activity in the muscles suggesting that the main target of this drug is the sarco-KATP channel rather than the mito-KATP channel. Previous works have shown that in isolated rat cardiac mitochondria, glimepiride fails to block the effects of KATP channel opening by GTP or diazoxide, in contrast to the blockage caused by glibenclamide, suggesting that this drug does not interfere with mitochondrial functions (Mocanu et al. 2001).

The KATP channel block and the atrophy induced by the drugs observed in the FDB fast-twitch fibers in our experiments resemble the condition of the age-dependent atrophy of the skeletal muscle apparatus specifically affecting fast-twitching muscles. In rat, the observed reduction in the KATP conductance parallels the reduction in the functional and metabolic demand of the muscles with aging and this may help to preserve the fast-twitch muscle from damage during voluntary contraction (Tricarico and Conte Camerino 1994; Pierno et al. 2013). Channel closure may therefore result in a safety valve for the fibers leading to apoptotic cell death and atrophy instead of necrotic cell death.

The atrophy induced in the SOL fibers by the sulfonylureas and glinides or by the 5-HD was related with the SDH activity measured in the same muscles indicating that the mitochondria plays a major role as atrophic pathway leading to a significant activation of the caspase 3 in this muscle phenotype. The order of efficacy of these drugs in inducing atrophy in SOL muscle was repa.≥glib.>nate.≥tolb.>glimep. which is similar to that observed in enhancing the SDH activity in this muscle type based on the one-way analysis of variance and it was repa.≥glib.>nate.≥tolb.>glimep. The involvement of the mitochondrial pathway has been shown in isolated mitochondria from rat skeletal muscle following treatment with glibenclamide and related drugs and it is believed to be responsible for the sulfonylureas-induced apoptosis (Skalska et al. 2005). The SOL muscle phenotype is indeed composed of slow-twitch type I oxidative fibers, which are characterized by a high mitochondrial density. These fibers also showed a reduced sarcolemma KATP currents in our experiments in mouse skeletal muscle as reported in rat (Tricarico et al. 2006, 2010). The involvement of the mitochondria as an atrophic pathway in SOL muscle has been confirmed by the fact that the 5-HD, which is a mito-KATP channel interacting drug, in our experiments was more effective as an atrophic agent in the SOL and FDB muscles showing an elevated mitochondrial density rather than in EDL muscle.

The molecular composition of the mito-KATP was initially identified into the SUR1/Kir6.1 complex. The mito-KATP complex is activated by diazoxide and inhibited by 5-hydroxydecanoate and included succinate dehydrogenase, which is inhibited by diazoxide, mitochondrial ATP-binding cassette protein-1 (mABC-1), ATP synthase, adenine nucleotide translocase, and phosphate carrier proteins, but it is not clear which component should be forming the channel pore. Lack of confirmed presence of canonical SUR or Kir6 subunits in mitochondria has led to alternative hypotheses regarding mito-KATP structure. Most recently, proteomic analysis of purified bovine mitochondrial inner membranes identified a short-form product of the KCNJ1 gene (ROMK2) as containing an N-terminal mitochondrial targeting signal and colocalization of a full-length epitope-tagged ROMK2 with mitochondrial ATP synthase β which imply a role for ROMK2 (Kir1) subunits in generating the mito-KATP channel (Nichols et al. 2013).

The observed low response of the EDL muscles to the sulfonylureas and 5-HD may be therefore related with the reduced activity of the surface KATP channels as observed in our experiments and reported also in rat EDL muscle (Tricarico et al. 2006). This is a fast-twitch glycolytic muscle composed of a mixed fiber population about 80–90% type IIB fast glycolytic and 10-20% type I oxidative fibers showing a reduced mitochondrial density with respect to the SOL.

The muscle phenotype seems to play a role in the atrophy induced by sulfonylureas and glinides; FDB muscle in this animal species is indeed composed of type I, IIA and IIX, the latter two being dominant, while EDL is primarily composed of fibers expressing IIB (60%) and IIX (60%) myosin, total over 100% because of hybrid myosin expression, and SOL mostly type I oxidative fibers. This would suggest that type I and IIA or IIX fibers may be more sensitive than pure fast-twitch glycolitic IIB fibers.

It should be stressed that some of the observed effects can be due to the interaction of the sulfonylureas or glinides on off targets present in the membrane patches or in the isolated fibers. For instance, sulfonylureas at high concentration may also interact with Kir6.2 subunit and this may contribute to the observed effects in our experiments (Gribble and Reimann 2003). On the other hand, the use of native muscle fibers is necessary to investigate on the relationships between the sarcolemma KATP channel activity recorded in different skeletal muscle phenotypes in a complex pathophysiological process such as atrophy.

### Clinical impact

Glyburide/glibenclamide show atrophy in humans during the 8 months of observation. A significant proportion of the muscle atrophy signal was observed with glyburide/glibenclamide in humans with respect to the tolbutamide or glimepiride. Case report analysis revealed that the patients suffering from muscle atrophy associated to glyburide use aged ≥65 years (*n* = 4 patients), were male, and were treated with polytherapy. In all reported cases, the glyburide was considered as a concomitant drug associated with other drugs. In one case, simvastatin, a well-known myotoxic drug, was associated with glyburide/glibenclamide. There were two cases of hospitalizations or prolongations of hospitalization not necessarily due to the muscle atrophy, per se. No case of overdose was associated with atrophy in these case reports. In FDA-AERS, it is not possible to evaluate the duration of concomitant drug therapy or the prescribed indication which are normally referred to the primary suspected drug. Despite the muscle atrophy is a common condition in the diabetic patient population, the PRR factor represents the risk to observe an atrophic event with a certain drug as compared with the other drugs of the same class.

We have, however, observed that equipotent compounds in “in vitro” experiments such as glibenclamide and repaglinide fail to result in the same number of adverse reports in humans. The investigation of the number of hypoglycemia cases for all drugs under investigation showed that glibenclamide is the most potent compound in inducing hypoglycemia in human. Taking into account the fact that hypoglycemia per se is capable to induce “in vitro” apoptosis and autophagic cell death, these observations suggest that high rates of hypoglycemia characterizing glibenclamide use are a precipitating factor in inducing atrophy “in vivo” in human (De la Cadena et al. 2013; Xiao et al. 2013). We should stress that the hypoglycemia rates observed with sulfonylureas and glinides are related with their PK properties. Glibenclamide is long-acting and usually taken 2x/day while repaglinide has a short half-life and is recommended to be taken with meals and the lower frequency of hypoglycemia is attributed to the repaglinide being cleared more rapidly than SU's and not a result of potency/selectivity. This becomes evident and relevant when comparing potency “in vitro” in our atrophy assays: repaglinide≥glibenclamide but repaglinide < glibenclamide in patients.

A limitation of our observation is that the actual plasma levels of glibenclamide are very likely far off the theoretically calculated 23 *μ*mol/L concentration which is the absolute maximum and that the actual concentration is likely significantly lower. This is due to the fact that volume of distribution of this drug will not equal the blood volume, glibenclamide is indeed very lipophilic and likely to accumulate in fat tissue thereby increasing the volume of distribution (that's used for estimating plasma exposure) significantly.

In conclusion, glibenclamide has the potential to induce atrophy “in vitro” in animal experiments and in human patients. Within the sulfonylureas investigated here glimepiride shows a less potential of inducing atrophy in either animal experiments or human patients. It should be stressed that the FDA considers glibenclamide not primarily responsible for atrophy. Rechallenge or dechallenge data would be helpful to establish a casual relationship between the drug and the signal but were not available. Therefore, the atrophic signal associated with this drug is weak. Based exclusively on clinical data, we cannot conclude that glibenclamide is an atrophic agent but combining the human data with the pharmacological data we can at least conclude that glibenclamide potentially exerts atrophic effects in skeletal muscle. This is why we indicated glibenclamide as a mild atrophic agent as compared with dexamethasone which is known to induce atrophy in human and in muscle cell lines (Dirks-Naylor and Griffiths 2009; Valiyil and Christopher-Stine 2010; Bonaldo and Sandri 2013).

Our data may have relevance in improving appropriate prescription drug use of best practice in patients affected by other neuromuscular disorders and diabetes or insulin/glucose dismetabolism.

## Abbreviations

Ac-DEVD-pNA: acetyl-Asp-Glu-Val-Asp-*p*-nitroaniline
ADR: adverse drug reactions
BSA: body surface area
DMEM+: Dulbecco's modified Eagle's medium
DMSO: dimethyl sulfoxide
EDL: extensor digitorum longus
EGTA: ethylene-bis(oxyethylenenitrilo)tetraacetic acid
FBS: fetal bovine serum
FDA-AERS: Food and Drug Administration-Adverse Effects Reporting System
FDB: flexor digitorum brevis
glib.: glibenclamide/glyburide
glimep.: glimepiride
HEPES: (4-(2-hydroxyethyl)-1-piperazineethanesulfonic acid)
KATP: ATP-sensitive K^+^ channel
HEPES: (4-(2-hydroxyethyl)-1-piperazineethanesulfonic acid )
Kir: inwardly rectifying K^+^ channel
mABC-1: mitochondrial ATP-binding cassette protein-1
MHC: myosin heavy chain
Mops: morpholino propanesulfonate
nate.: nateglinide
PRR: proportional reporting ratio
repa.: repaglinide
SDH: succinic dehydrogenases.
SOL: soleus
SUR: sulfonylurea receptor
tolb.: tolbutamide
